# Evaluation of the Direction of External Force Input to the Skull and Its Influence on the Severity of Traumatic Brain Injury

**DOI:** 10.7759/cureus.95781

**Published:** 2025-10-30

**Authors:** Mizuki Mori, Yuto Aramaki, Kazunori Fukushima, Kiyohiro Oshima

**Affiliations:** 1 Department of Emergency Medicine, Gunma University Graduate School of Medicine, Maebashi, JPN

**Keywords:** abbreviated injury scale, acute subdural hematoma, computed tomography, craniotomy, frail elderly, intracranial hemorrhage, prehospital emergency care, skull fractures, talk and die syndrome, traumatic subarachnoid hemorrhage

## Abstract

Background: Recent demographic changes in Japan have led to an increasing incidence of traumatic brain injury (TBI) resulting from low-energy mechanisms such as falls among the elderly. However, the influence of the direction of external force input on the severity of head injury has not been well characterized in clinical cohorts. This study aimed to evaluate the relationship between the direction of cranial impact and the severity of TBI, with particular attention to the anatomical fracture site and the need for emergency neurosurgical intervention.

Methods: This retrospective cohort study included 231 patients with blunt head trauma admitted to Gunma University Hospital between April 2018 and March 2023. The impact direction was classified as longitudinal or transverse according to injury patterns and prehospital documentation. Skull fractures were categorized into five anatomical regions: frontal, parietal, occipital, temporal, and basilar skull. The primary outcomes were the occurrence of severe TBI, defined as head AIS ≥ 5, and the performance of emergency craniotomy. Multivariate logistic regression analyses were performed to estimate odds ratios (ORs) and 95% confidence intervals (CIs), controlling for age and antithrombotic use. Model calibration was verified using the Hosmer-Lemeshow test, and sensitivity analysis was conducted by comparing minimally adjusted and primary models.

Results: Among the included patients, 70% sustained injuries due to falls, and more than half were aged ≥ 65 years. The proportion of severe TBI was higher in the transverse impact group (23%) than in the longitudinal group (13%). Temporal and basilar skull fractures were independently associated with severe TBI (temporal: OR 3.84, 95% CI 1.63-9.05; P = 0.002; basilar: OR 7.61, 95% CI 2.78-20.9; P < 0.001) and emergency craniotomy (temporal: OR 3.13, 95% CI 1.29-7.16; P = 0.011; basilar: OR 2.90, 95% CI 1.00-8.36; P = 0.049). No significant group differences were observed in mortality at 14, 30, or 180 days. The overall in-hospital mortality rate was 18.2% (42/231), comparable to previously reported rates in moderate-to-severe TBI cohorts. The median Glasgow Outcome Scale-Extended (GOSE0 at 180 days did not differ significantly between groups (P = 0.289), although survivors in the transverse group had better functional outcomes (P = 0.042).

Conclusions: Temporal and basilar skull fractures, as well as transverse impact mechanisms, are associated with higher injury severity and a greater likelihood of emergency craniotomy but are not independent predictors of mortality. These findings suggest that the direction of cranial impact provides important biomechanical insight into injury severity and may aid in prehospital triage and preventive strategies for head trauma in aging populations.

## Introduction

According to the Japan Trauma Data Bank 2022, traumatic brain injuries (TBIs) represent the second most common type of trauma in Japan, following lower extremity injuries, and account for approximately one-third (33.0%) of all trauma cases [1]. Moreover, head injuries remain the leading cause of trauma-related mortality, responsible for 43.3% of all trauma deaths reported in the 2023 Vital Statistics [2]. Globally, TBI is a major cause of death and disability among young individuals in developed nations [3]. Even when survival is achieved, persistent higher brain dysfunction may result in profound long-term disability and socioeconomic burden. Therefore, predicting and preventing severe TBI constitutes a critical priority in emergency medicine.

Previous studies, particularly in the context of automotive safety research, have proposed several biomechanical indices for estimating head injury severity, including the Head Injury Criterion (HIC) [4], Rotational Injury Criterion (RIC) [5], and Cumulative Strain Damage Measure (CSDM) [6]. Furthermore, the use of finite element models has enabled simulation of vehicle collisions to estimate the von Mises strain within brain tissue, and such methodologies have contributed to improvements in vehicle safety design [7].

However, these indices primarily address controlled experimental conditions involving high-energy impacts, such as those sustained in motor vehicle collisions or contact sports, rather than the mechanisms most frequently encountered in community settings. In everyday clinical practice, particularly in Japan, the majority of head injuries result from low-energy events such as simple falls or tumbles. Although these mechanisms are generally perceived as minor, a subset of patients, especially older adults, initially present with mild symptoms but subsequently deteriorate rapidly, often necessitating emergency surgical intervention. This phenomenon, commonly referred to as the “Talk and Deteriorate” [8], underscores the challenge of early identification of high-risk patients. However, the biomechanical directionality of such low-energy injuries has not been well characterized in clinical cohorts.

While prior research has predominantly focused on high-velocity or high-impact injuries, these models may not accurately represent the actual clinical spectrum of head trauma in Japan, where the aging population and the predominance of fall-related injuries shape the epidemiological landscape. The specific biomechanical characteristics-particularly the direction of cranial impact-that predispose patients to severe outcomes remain insufficiently elucidated.

Therefore, the present study aimed to investigate the relationship between the direction of external force applied to the cranium and the severity of TBI, with a focus on identifying impact patterns associated with high-risk injuries. Clarifying these associations may enable earlier recognition of potentially life-threatening cases in the prehospital phase and contribute to improved triage and prevention strategies for elderly and vulnerable populations.

## Materials and methods

Study design and settings

This retrospective, single-center cohort study was conducted at Gunma University Hospital, Japan. The study protocol was reviewed and approved by the Institutional Review Board of Gunma University Hospital (Approval No. IRB 2023-044 (2115)). Owing to the retrospective nature of the investigation, the requirement for written informed consent was waived. All procedures were performed in accordance with the ethical standards of the Declaration of Helsinki.

Gunma University Hospital is in Maebashi City (the population was approximately 329,000 in 2023, with adults aged 65 years or older comprising 30.2% of the population [9] ) which is the prefectural capital in Gunma Prefecture (population of 1.9 million [10], 31.2% of which are adults aged 65 years or older [11] ) and is located in a regional urban center encompassing both urban and mountainous rural areas. As a regional core medical center, our hospital consistently accepts and treats a wide range of patients from Gunma Prefecture and its neighboring prefectures; thus, patients at our hospital reflect Japan’s diverse geographic and demographic characteristics. Our hospital certainly treats a wide range of head trauma cases, including mild to severe injuries, as well as cases involving children and pregnant women. Therefore, our hospital was considered to be an appropriate setting for estimating the study population.

The study period spanned five years, from April 1, 2018, to March 31, 2023. All patients admitted to the department of emergency medicine or neurosurgery with blunt head trauma requiring hospitalization were screened for eligibility. Clinical data were obtained from electronic medical records, emergency medical service (EMS) transport reports, and air medical (helicopter) operation logs. The study adhered to the Strengthening the Reporting of Observational Studies in Epidemiology (STROBE) guidelines for observational cohort studies.

Definitions and variables

All patients admitted with blunt head trauma requiring hospitalization during the study period were initially screened for eligibility. Inclusion criteria were as follows: (1) positive findings on head computed tomography (CT) confirming skull fracture or intracranial injury, and (2) availability of sufficient documentation to estimate the direction of external force applied to the skull. Exclusion criteria included: (1) penetrating cranial trauma, (2) unclear or indeterminate mechanism of injury, (3) missing or incomplete imaging data that precluded classification, and (4) inadequate prehospital records or unavailable EMS documentation.

**Figure 1:**
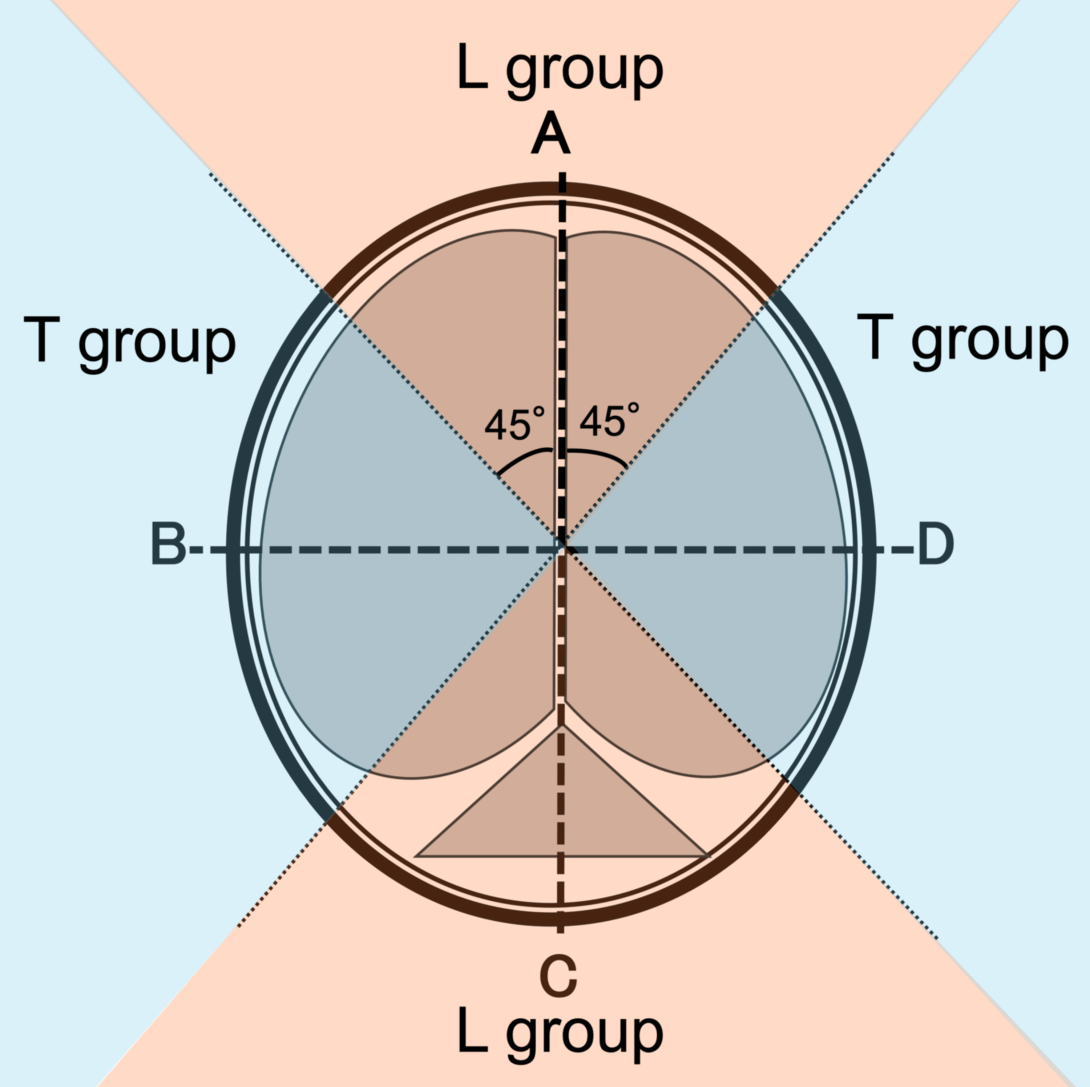
Definition of the direction of external force input to the cranium This is a schematic diagram of a horizontal section of the cranium. Cases with external forces input from areas A and C were classified into the longitudinal group (L group), and cases with external forces input from areas B and D were classified into the transverse group (T group). The figure is the authors' own creation.

The direction of external impact was classified as either transverse or longitudinal depending on the primary vector of force transmission relative to the cranial axis. Transverse impacts were defined as lateral or side-to-side forces transmitted across the skull, typically involving the temporal or parietal regions. Longitudinal impacts were defined as vertical or anteroposterior forces directed along the cranial midline, commonly affecting the frontal or occipital regions. The direction of external impact was categorized as transverse or longitudinal according to the primary vector of force transmission relative to the cranial axis (Figure 1).

Operational definitions were applied as follows: transverse denoted lateral, or side-to-side transmission, typically involving temporal/parietal regions; longitudinal denoted vertical or anteroposterior transmission commonly affecting frontal/occipital regions. Classification relied on a composite review of (i) external injury morphology (lacerations/contusions/hematomas), (ii) skull fracture pattern and anatomical site on CT, and (iii) prehospital photographs and written EMS documentation (e.g., spider-web-like windshield cracks or bonnet dents indicating head or torso impact [12]).

At our center, initial classification is performed by emergency physicians who simultaneously consult neurosurgeons. Each case undergoes contemporaneous joint review by the emergency physician and a board-certified neurosurgeon with direct access to identical imaging and prehospital source documents. Any discrepancy or uncertainty triggers immediate consensus adjudication, during which reviewers re-examine raw images and clinical notes to reach a shared determination. This prospective, consensus-based, dual-specialty workflow was instituted to enhance procedural objectivity and minimize misclassification without delaying time-critical care. Because independent parallel readings were not archived, we did not compute inter-rater statistics (e.g., Cohen’s κ). To mitigate potential bias, explicit operational criteria were used, and cases with persistent ambiguity regarding direction were excluded a priori from direction-specific analyses (see exclusion criteria).

**Figure 2:**
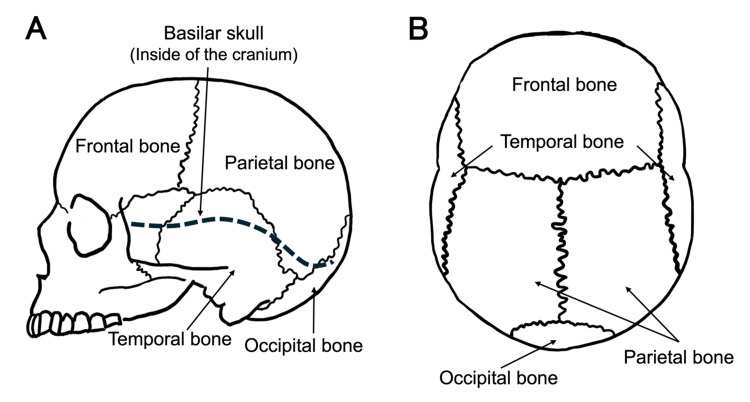
Classification and definition of skull fractures Panel A is a schematic representation of the skull viewed from the lateral aspect, while Panel B shows the skull from the superior aspect. The figures are the authors' own creations.

Clinical variables included demographic data (age, sex), mechanism of injury, and use of antithrombotic medication. The neurological status at admission was assessed by the Glasgow Coma Scale (GCS). Radiological variables comprised the type and location of skull fractures and the presence of intracranial lesions, including acute subdural hematoma (ASDH), acute epidural hematoma (AEDH), traumatic subarachnoid hemorrhage (tSAH), cerebral contusion, and diffuse axonal injury (DAI). Outcome variables included the need for emergency craniotomy or craniectomy, in-hospital mortality, and functional outcome as measured by the Glasgow Outcome Scale-Extended (GOSE) [13] at 180 days post-injury. For further risk stratification, skull fractures were classified into five anatomical regions: temporal, parietal, occipital, frontal, and basilar skull (Figure 2).

These skull components are anatomically delineated by sutures. Although the skull base includes several bones (frontal, occipital, temporal, sphenoid, and ethmoid), it is clinically referred to as the basilar skull, and this classification was adopted in our study. Injury mechanisms were categorized as follows: trauma due to motor vehicle accidents included collisions with passenger cars, trucks, motorcycles, or trains operated by third parties, while trauma from falls or tumbles involved self-inflicted injuries without third-party participation. Emergency craniotomies were defined as unscheduled neurosurgical interventions performed in response to increasing intracranial hematomas or impending brain herniation, including hematoma evacuation, burr hole surgery, decompressive craniectomy, or decompressive internal procedures. At Gunma University Hospital, neurosurgical emergency procedures are available 24 hours a day.

The severity of head trauma was assessed using the Abbreviated Injury Scale (AIS) [14]. Head AIS scores are primarily determined by hemorrhage volume (hematoma thickness), whether contusions are superficial or deep, and the presence of persistent coma. Based on previous studies [15], AIS scores of 1-2 were classified as mild, scores of 3-4 were classified as moderate, and scores of 5-6 were classified as severe.

Outcomes

The primary outcome of this study was the severity of TBI, assessed using the Abbreviated Injury Scale (AIS) for the head region. The study aimed to determine whether the direction of external impact (transverse vs. longitudinal) and the anatomical location of skull fracture were associated with increased head AIS scores, reflecting more severe intracranial injury.

Secondary outcomes included (1) the necessity of emergency craniotomy, defined as unscheduled neurosurgical intervention for expanding hematoma or impending herniation; (2) in-hospital mortality; and (3) functional outcomes at 180 days after injury, assessed by the GOSE. These secondary outcomes were analyzed to investigate whether the direction of external impact or fracture distribution influenced clinical course or long-term recovery. GOSE had eight categories: death, vegetative state, (lower/upper) severe disability, (lower/upper) moderate disability, and (lower/upper) good recovery. Follow-up was conducted using hospital records and, for transfers, medical correspondence with the receiving facility.

Statistical analysis

Descriptive statistics were used to evaluate the distribution of variables. Continuous variables were summarized using medians and interquartile ranges (IQRs), while categorical variables were presented as counts and percentages. Group comparisons were performed using the Chi-square test or Fisher’s exact test for categorical data, and the Mann-Whitney U test was conducted for continuous data. Multivariate logistic regression analysis was conducted to estimate the odds ratios and 95% confidence intervals for the occurrence of severe traumatic brain injury. The covariates included in the model were age and regular use of antithrombotic agents.

These covariates were selected because age-related decline in physical function, such as impairments in vision, hearing, balance, muscle strength, joint disease, or cognitive ability, is a known risk factor for head trauma [16]. In elderly patients aged 60-65 years or older, brain atrophy leads to enlargement of the subdural space, which increases the buffering capacity against mechanical forces. As a result, rotational forces may cause less shear stress on the brain parenchyma, and symptoms may initially be absent [17]. Consequently, conventional CT criteria may overlook brain injury [18]. On the other hand, intracranial compliance is reduced in the elderly, leading to rapid and severe deterioration once decompensation occurs [19]. According to reports, for every 10-year increase in age, the odds of poor outcomes increase by 40-50% [20]. In addition, up to 25% of elderly patients undergoing anticoagulation therapy may develop intracranial hemorrhages, even when presenting with mild TBI and a Glasgow Coma Scale score of 15 [21]. Some patients in this group may develop delayed acute subdural hematomas (ASDHs) within 24-96 hours post-injury, even if no abnormalities are detected on the initial CT scan [22]. These factors necessitate careful consideration of age and antithrombotic therapy as confounding variables.

All hypothesis testing was two-sided, and a P-value < 0.05 was considered to be statistically significant. All statistical analyses were conducted using EZR (Saitama Medical Center, Jichi Medical University, Saitama, Japan) [23], which is a graphical user interface for R (The R Foundation for Statistical Computing, Vienna, Austria). EZR is a modified version of R Commander that is designed to incorporate statistical functions frequently used in biostatistics. Data completeness was verified for all major variables included in the multivariate models. Information on age, sex, impact direction, fracture site, and TBI diagnosis was complete for all cases.

Antithrombotic use was missing in 16 of 231 patients (6.9%). As the absence was considered random, these cases were excluded from the multivariate analyses by listwise deletion. The relatively small proportion of missing data was unlikely to introduce substantial bias. Laboratory parameters and GOSE scores had limited missing data, which were not included in the regression models. These variables were summarized descriptively based on available cases, and the number of missing values was indicated in the corresponding table footnotes. Model calibration was assessed using the Hosmer-Lemeshow goodness-of-fit test, and multicollinearity was evaluated using variance inflation factors (VIFs).

## Results

**Figure 3:**
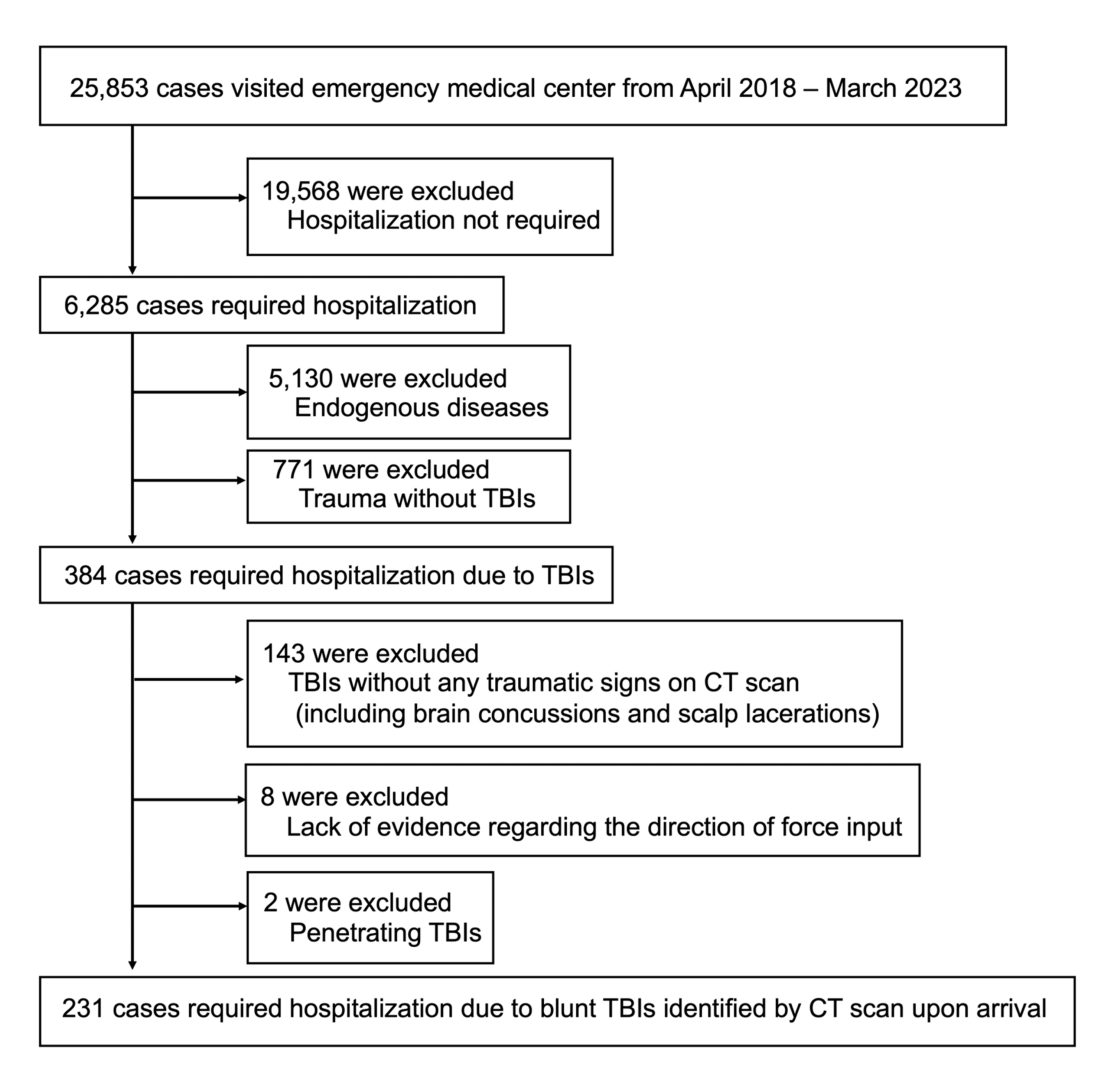
Identification and classification of the patients There were no patients with multiple admissions during the study period. CT: computed tomography, TBI: traumatic brain injury. The figure is the authors' own creation.

A flow diagram of patient inclusion is presented in Figure 3.

A total of 231 patients were included in the final analysis after applying the inclusion and exclusion criteria. The demographic and clinical characteristics of patients in the lateral (L) and transverse (T) impact groups are summarized in Table 1.

**Table 1:** Comparisons of clinical characteristics between L and T groups Bold P-value: Statistically significant at p < 0.05. Group comparisons were performed using the Chi-square test or Fisher’s exact test for categorical data, and the Mann-Whitney U test was conducted for continuous data. 1: Cases longer than 24 hours were excluded as outliers, 2: Multiple trauma was defined as trauma to two or more sites with an AIS score 3 or higher, 3: Allowing duplication within the same case, 4: Laboratory data were unavailable in 63 cases (27.3%, FDP), 77 cases (33.3%, fibrinogen), 10 cases (4.3%, hemoglobin), 11 cases (4.3%, platelet), 41 cases (17.7%, lactate). Percentages are based on available data, 5: Eight (8) corresponds to full recovery and one (1) corresponds to death. GOSE scores were missing in 109 cases (47.2 %). Percentages are based on available data. L: lateral, T: transverse, IQR: interquartile range, GCS: Glasgow Coma Scale, SBP: systolic blood pressure, RR: respiratory rate, AIS: Abbreviated Injury Scale, tSAH: traumatic subarachnoid hemorrhage, ASDH: acute subdural hematoma, AEDH: acute epidural hematoma, ISS: Injury Severity Score, FDP: fibrinogen/fibrin degradation products, GOSE: Glasgow Outcome Scale Extended, TBI: traumatic brain injury.

Characteristic			L group (N=134)	T group (N=97)	Test Statistic	P-value
Age	─yr, Median (IQR)		70.5 (55.25-79)	72 (51-81)	U = 6,611	0.824
	Age group ─no. (%)	0-17 yr	9 (7)	7 (7)	χ^2 ^= 0.554	0.758
		18-64 yr	52 (39)	33 (34)		
		≧65 yr	73 (54)	57 (59)		
Sex; Male ─no. (%)			96 (72)	62 (64)	χ^2 ^= 1.216	0.270
Currently use any antithrombotics ─no. (%)			24 (18)	21 (22)	χ^2 ^= 0.291	0.589
Injured in fall or tumble ─no. (%)			95 (71)	71 (73)	χ^2 ^= 0.055	0.814
Elapsed time ─min, Median (IQR)^※^^1^	Injury to hospital		42.5 (32.75-73)	42 (33-59.5)	U = 4053.5	0.630
	Injury to CT scan		81 (60-116.5)	72.5 (56-96)	U = 4335.5	0.108
Vital signs on arrival ─Median (IQR)	GCS		14 (7-15)	14 (12-15)	U = 6,029	0.333
	SBP		138 (113.8-163)	139 (116-168)	U = 6,030	0.720
	RR		17 (12-20)	18 (14-21)	U = 5,936	0.493
Anisocoria ─no. (%)			15/130 (11.5)	12/95 (12.7)	χ^2 ^= 0.002	0.967
Multiple injuries ─no. (%)^※^^2^			19 (14)	19 (19.6)	χ^2 ^= 0.836	0.360
	TBIs as Max AIS		16 (11.9)	14 (14.4)	χ^2 ^= 0.128	0.720
ISS score ─Median (IQR)			9 (4-17)	16 (9-25)	U = 5,164	0.007
Head AIS score ─Median (IQR)			3 (2-4)	3 (2-4)	U = 5,407	0.023
	Severity ─no. (%)	mild	56 (42)	29 (30)	χ^2 ^= 5.028	0.081
		moderate	60 (45)	46 (47)		
		severe	18 (13)	22 (23)		
Focal brain injury ─no. (%) ^※^^3^	tSAH		30 (22)	56 (58)	χ^2 ^= 28.585	<0.001
	ASDH		18 (13)	42 (43)	χ^2 ^= 24.573	<0.001
	Contusion		11 (8)	14 (14)	χ^2 ^= 1.660	0.198
	AEDH		12 (9)	9 (9)	χ^2 ^= 5.552	1
Cranial fracture ─no. (%) ^※^^3^	Temporal bone		19 (14)	33 (34)	χ^2 ^= 11.587	<0.001
	Parietal bone		20 (15)	18 (19)	χ^2 ^= 0.308	0.579
	Occipital bone		24 (18)	12 (12)	χ^2 ^= 0.925	0.336
	Frontal bone		28 (21)	6 (6)	χ^2 ^= 8.563	0.003
	Basilar skull		20 (15)	12 (12)	χ^2 ^= 0.131	0.718
Initial laboratory data ─Median (IQR) ^※^^4^	FDP ─ μg/mL		57.9 (17.55-223.4)	44.5 (13.7-228.4)	U = 3,721	0.524
	Fibrinogen ─ mg/dL		235 (190.25-278)	269 (195-319)	U = 3,776	0.005
	Hemoglobin ─ g/dL		13.2 (11.7-14.8)	12.95 (11.5-14.1)	U = 6,689	0.135
	Platelet ─ 10^4^/μL		20.45 (15.875-23.85)	19.7 (15.625-24.875)	U = 7,035	0.017
	Lactate ─ mmol/L		2.25 (1.4-4.15)	1.9 (1.5-3.575)	U = 4,148.5	0.380
Performed craniotomy ─no. (%)			10 (7.5)	20 (20.1)	χ^2 ^= 7.493	0.006
Hospital stay ─Day, Median (IQR)			9.5 (4-26)	9 (3-22)	U = 6694.5	0.697
Death ─no. (%)	In-hospital death		27 (25.2)	15 (18.3)	χ^2 ^= 0.545	0.460
	Day 1-14		26/117 (22.2)	14/78 (17.9)	χ^2 ^= 0.295	0.587
	Day 1-30		26/108 (24.1)	15/69 (21.7)	χ^2 ^= 0.031	0.860
	Day 1-180		28/78 (35.9)	17/52 (32.7)	χ^2 ^= 0.035	0.851
GOSE score (on Day 180) ─Median (IQR)^※^^5^	Overall patients		4 (1-7.25)	6.5 (1-8)	U = 1,603.5	0.289
	Survivors		7 (6-8)	8 (7-8)	U = 513.5	0.042

More than half of the patients in both groups were aged 65 years or older, and most were male. Approximately 70% of injuries resulted from falls or tumbles. Regarding imaging workflow, although there was variation in the time from injury to CT due to inter-facility transfers, CT evaluation was generally completed within 30 minutes after arrival. The proportion of severe cases (AIS ≥ 5) was higher in the T group (23%) than in the L group (13%). The T group also exhibited markedly higher median AIS scores for the head and overall Injury Severity Scores compared to the L group. tSAH and ASDH were markedly more frequent in the T group (tSAH: 58% vs. 22%, P < 0.001; ASDH: 43% vs. 13%, P < 0.001). Temporal bone fractures were more frequent in the T group, while frontal bone fractures predominated in the L group. The proportion of patients undergoing emergency craniotomy was also markedly higher in the T group (20% vs. 7.5%, P = 0.006).

No significant differences were observed between groups in hospital length of stay or mortality at 14, 30, or 180 days. The median GOSE score at 180 days indicated moderate to severe disability in both groups (L group: 4 (IQR, 1-7.25); T group: 6.5 (IQR, 1-8); P = 0.289). The results from the multivariate logistic regression analysis based on cranial fracture site are shown in Table 2.

**Table 2:** Relationship between skull fracture and risk of occurrence of severe head injury Bold P-value: Statistically significant at p < 0.05. AIS: Abbreviated Injury Scale, CI: confidence interval, OR: odds ratio from multivariable logistic regression. Confounders adjusted for: age and regular use of antithrombotic agents.

Type of fracture	AIS score≧5			Performed craniotomy		
	OR	95%CI	P-value	OR	95%CI	P-value
Total	2	0.89-4.49	0.093	2.16	0.93-5.03	<0.001
Frontal bone	0.87	0.28-2.69	0.801	0.69	0.19-2.46	0.569
Temporal bone	3.84	1.63-9.05	0.002	3.13	1.29-7.16	0.011
Parietal bone	3.13	1.19-8.23	0.021	0.74	0.20-2.69	0.645
Occipital bone	2.99	1.12-7.95	0.282	2.44	0.89-6.69	0.083
Basilar skull	7.61	2.78-20.9	<0.001	2.9	1.00-8.36	0.049

Temporal bone fracture was independently associated with the occurrence of severe TBI (OR, 3.84; 95% CI, 1.63-9.05; P = 0.002). Basilar skull fracture also showed a strong association with severe TBI (OR, 7.61; 95% CI, 2.78-20.9; P < 0.001). The same fracture sites were significantly correlated with the performance of emergency craniotomy (temporal bone: OR 3.13, 95% CI 1.29-7.16, P = 0.011; basilar skull: OR 2.90, 95% CI 1.00-8.36, P = 0.049). No significant associations were found for frontal, parietal, or occipital bone fractures.

Model calibration was assessed for each logistic regression model using the Hosmer-Lemeshow goodness-of-fit test, which indicated no significant lack of fit (P = 0.16-0.92 across models); no multicollinearity was detected (VIF = 1.0-1.1). In the sensitivity analysis comparing the minimally adjusted and primary models, the effect estimate for transverse impact on severe TBI showed moderate variation (minimally adjusted model: OR 2.68, 95% CI 1.26-5.67; primary model: OR 2.00, 95% CI 0.89-4.49). However, the direction and overall interpretation of the association remained consistent. Thus, the findings were considered robust to model specification. For emergency craniotomy, the effect estimates were nearly identical between models.

The overall in-hospital mortality rate was 18.2% (42/231). Mortality did not differ significantly between patients with and without temporal or basilar skull fractures (P = 0.746). Similarly, when stratified by the direction of external impact, patients with transverse impacts did not show a significantly higher mortality rate compared with those with longitudinal impacts (p = 0.460). These findings suggest that, although temporal and basilar fractures were associated with more severe intracranial injuries, their presence and the direction of impact were not independent predictors of in-hospital mortality in this cohort.

Functional outcomes at 180 days post-injury were assessed using the GOSE. Among survivors, the median GOSE score was 7 (IQR, 6-8). When all cases, including fatalities, were analyzed, there was no significant difference in GOSE score between the transverse and longitudinal impact groups (P = 0.289). However, among survivors only, the transverse impact group demonstrated markedly better functional outcomes compared with the longitudinal group (P = 0.042).

## Discussion

Recent epidemiological data in Japan demonstrate a steady decline in motor vehicle-related head injuries among car or motorcycle occupants, whereas the incidence of head trauma in pedestrians and elderly individuals caused by falls or tumbles has increased [24-26]. The annual number of trauma patients aged 65 years or older has been rising since 2015, while a slight increase has also been observed among younger individuals aged 15-19 years [25]. According to the White Paper on Traffic Safety, traffic accidents and related deaths have both markedly decreased since their peak in 2004; fatalities within one month of injury had dropped to 41.9% of 1998 levels by 2015 [27]. Despite these improvements, outcomes among the elderly remain poor, with an increasing proportion of severe disability and vegetative states compared with younger age groups [28]. Given these demographic changes, trauma management in Japan must increasingly address low-energy mechanisms and age-specific physiological vulnerabilities. As the world’s most aged society [29], Japan faces an urgent need to develop strategies for both injury prevention and post-injury functional recovery.

The cornerstone of initial management for TBI lies in stabilizing systemic physiology and preventing secondary injuries resulting from hypoxia or coagulopathy, in addition to mitigating the primary mechanical insult [30]. In this study, 79% (30/38) of patients with multiple trauma exhibited the most severe injury (Max AIS) in the head region, consistent with previous findings that head trauma is a major determinant of mortality and morbidity in multiple trauma [31]. Identifying high-risk mechanisms of cranial impact could enable early recognition of injuries likely to require urgent intervention, even before hospital arrival, based on traffic camera footage or emergency medical service (EMS) observations, and inform both prehospital triage and vehicle safety design.

The present study examined the relationship between the direction of external force applied to the skull, skull fracture location, and the severity and outcomes of TBI in a Japanese population. Our findings provide novel insight into how lateral and basal impact patterns contribute to intracranial injury characteristics, while also highlighting the complexity of predicting outcomes based solely on fracture distribution or impact direction.

Previous biomechanical and clinical studies have suggested that the direction of cranial impact influences intracranial strain propagation and lesion patterns. For instance, lateral or basal impacts may transmit shear forces through the middle cranial fossa and temporal bone, resulting in contusions or hematomas in the temporal lobe or brainstem. Conversely, longitudinal or frontal impacts are thought to produce more linear fracture lines and localized epidural or subdural hematomas [32,33]. In our cohort, fractures involving the temporal and basilar regions were associated with increased acute injury severity, consistent with these prior reports. The use of CT-based fracture mapping and impact direction analysis enabled a more detailed characterization of these relationships in low- to moderate-energy trauma, an aspect particularly relevant to Japan’s aging population.

However, despite the observed association between fracture sites and acute TBI severity, neither fracture location nor impact direction was independently related to in-hospital mortality in our multivariable analysis. The overall mortality rate of 18.2% (42/231) was comparable to previous reports of moderate-to-severe TBI among elderly or multiple trauma patients [34,35]. It is conceivable that improvements in prehospital triage, emergency transport systems, and acute neurosurgical management have mitigated the lethality historically associated with basal and temporal fractures.

Our data thus suggest that anatomical injury patterns alone cannot fully predict fatal outcomes, and that systemic factors, such as age, comorbidities, anticoagulant therapy, and circulatory instability, may play more dominant roles in determining survival. In contrast, analysis of long-term functional outcomes revealed an intriguing trend. Although no significant difference in GOSE was observed when all cases, including deaths, were analyzed, survivors in the transverse impact group showed markedly better functional recovery than those in the longitudinal group. One plausible interpretation is that transverse impacts tend to produce more focal lesions, such as temporal or basilar fractures, while relatively sparing diffuse axonal pathways responsible for persistent neurological disability. Once the acute phase is survived, these patients may thus exhibit better recovery potential. Nevertheless, given the retrospective and single-center design of this study, these observations should be interpreted cautiously. Larger, prospective multicenter studies are warranted to validate whether impact direction truly influences post-TBI functional trajectories.

Taken together, our results underscore that the direction of impact and fracture distribution primarily affect acute injury patterns, whereas long-term functional outcomes are more likely influenced by patient-level and systemic factors. Understanding these directional dynamics may assist clinicians in early risk stratification and in tailoring preventive strategies, particularly for elderly individuals at risk of low-energy falls.

This study has several limitations that should be acknowledged. First, its retrospective, single-center design inherently limits the generalizability of the findings and the ability to control for unmeasured confounders. Although the inclusion and exclusion criteria were clearly defined, selection bias cannot be entirely ruled out. Second, the direction of external force was determined primarily from prehospital documentation, external injuries, and imaging findings. While these assessments were verified through neurosurgical consultation at the time of admission, inter-observer variability could not be completely eliminated. Future studies employing standardized image-based classification or sensor-derived kinematic data may improve the objectivity and reproducibility of impact direction assessment. Third, the sample size was relatively modest, and certain analyses-particularly those related to fracture site and long-term outcome-may have been underpowered. Fourth, the functional outcome assessment (GOSE) was limited by incomplete follow-up data for some cases, which could have introduced attrition bias. Lastly, as this study focused on low- to moderate-energy trauma, its findings may not be directly applicable to high-velocity injuries such as traffic collisions.

Despite these limitations, the present study provides valuable insight into the relationship between impact direction, skull fracture pattern, and TBI severity in a population that reflects the growing burden of geriatric trauma in Japan. Understanding these directional and anatomical injury characteristics may help refine prehospital triage algorithms and inform preventive strategies for fall-related head injuries in the elderly. Future research should include multicenter, prospective studies incorporating biomechanical modeling and imaging-based validation to clarify how external force vectors influence the biomechanical pathways and clinical outcomes of TBI.

## Conclusions

In summary, this retrospective observational study demonstrated that the direction of external impact and the location of skull fractures were associated with the severity patterns of traumatic brain injury but were not independent predictors of in-hospital mortality. Although temporal and basilar skull fractures tended to accompany more severe intracranial lesions, neither the fracture sites nor the direction of impact significantly influenced survival. However, among survivors, those who sustained transverse impacts exhibited better functional recovery at 180 days compared with those exposed to longitudinal impacts.

These findings suggest that while the direction of impact and fracture distribution reflect the mechanisms of acute brain injury, long-term functional outcomes are more strongly determined by systemic factors and the capacity for neurological recovery. Further multicenter, prospective studies integrating biomechanical analysis and clinical validation are warranted to clarify how impact dynamics influence the severity and recovery potential of TBI.

## References

[1] Satoshi T (2023). Mild traumatic brain injury and mild blast-induced traumatic brain injury (Article in Japanese). J Natl Def Med Coll.

[2] (2025). Ministry of Health Labour and Welfare: Vital statistics of Japan Final data General mortality. https://www.e-stat.go.jp/stat-search/files?page=1&layout=datalist&toukei=00450011&tstat=000001028897&cycle=7&year=20230&month=0&tclass1=000001053058&tclass2=000001053061&tclass3=000001053065&stat_infid=000040206186&result_back=1&cycle_facet=tclass1%3Atclass2%3Atclass3&tclass4val=0.

[3] Levin HS, Diaz-Arrastia RR (2015). Diagnosis, prognosis, and clinical management of mild traumatic brain injury. Lancet Neurol.

[4] Versace J (1971). A review of the Severity Index. Society of Automotive Engineers Technical Paper.

[5] Kimpara H, Iwamoto M (2012). Mild traumatic brain injury predictors based on angular accelerations during impacts. Ann Biomed Eng.

[6] Takhounts EG, Eppinger RH, Campbell JQ, Tannous RE, Power ED, Shook LS (2003). On the development of the SIMon Finite Element Head Model. Stapp Car Crash J.

[7] Kisanuki Y, Sakuma S, Miki I, Matsuoka A, Hasegawa J, Yoshida S, Kono M (2002). Injury reconstruction in a traffic accident using total human model (Article in Japanese). Bioengineering Conference Proceedings.

[8] Yohtaro S, Yoshio T, Homare N, Hidetaka O, Takashi M, Masashi U (2016). Analysis of talk & deteriorate patients: report of our own cases and review of literature. Neurotraumatology.

[9] (2025). Maebashi City: Basic Resident Register Population Table by Age. https://www-city-maebashi-gunma-jp.translate.goog/soshiki/shimin/shimin/gyomu/4/26412.html?_x_tr_sl=ja&_x_tr_tl=en&_x_tr_hl=ja.

[10] (2025). Gunma Prefecture: 2023 Gunma Prefecture Population Survey Results (Annual Report). https://www.pref.gunma.jp.e.aag.hp.transer.com/page/632325.html.

[11] (2025). Gunma Prefecture: Population by age of Gunma Prefecture (Population Survey by Age of Gunma Prefecture / As of October 1, 2023). https://www.pref.gunma.jp.e.aag.hp.transer.com/page/621269.html.

[12] Shinichi I, Yasuo O (2023). Prediction of pedestrian’s head collision position by analysis of 56 accidents and mathematical model simulation (Article in Japanese). Jpn J Forensic Sci Technol.

[13] Wilson L, Boase K, Nelson LD (2021). A manual for the Glasgow Outcome Scale-Extended interview. J Neurotrauma.

[14] Gennarelli TA, Wodzin E (2006). AIS 2005: a contemporary injury scale. Injury.

[15] Savitsky B, Givon A, Rozenfeld M, Radomislensky I, Peleg K (2016). Traumatic brain injury: it is all about definition. Brain Inj.

[16] Maeda T, Katayama Y, Yoshino A (2018). Current status and issues of head injuries in the elderly (Article in Japanese). Jpn J Neurosurg (Tokyo).

[17] Karibe H, Hayashi T, Hirano T, Kameyama M, Nakagawa A, Tominaga T (2014). Characteristics and problems of head injuries in the elderly (Article in Japanese). Jpn J Neurosurg (Tokyo).

[18] Mack LR, Chan SB, Silva JC, Hogan TM (2003). The use of head computed tomography in elderly patients sustaining minor head trauma. J Emerg Med.

[19] Albeck MJ, Skak C, Nielsen PR, Olsen KS, Børgesen SE, Gjerris F (1998). Age dependency of resistance to cerebrospinal fluid outflow. J Neurosurg.

[20] Hukkelhoven CW, Steyerberg EW, Rampen AJ (2003). Patient age and outcome following severe traumatic brain injury: an analysis of 5600 patients. J Neurosurg.

[21] Reynolds FD, Dietz PA, Higgins D, Whitaker TS (2003). Time to deterioration of the elderly, anticoagulated, minor head injury patient who presents without evidence of neurologic abnormality. J Trauma.

[22] Itshayek E, Rosenthal G, Fraifeld S, Perez-Sanchez X, Cohen JE, Spektor S (2006). Delayed posttraumatic acute subdural hematoma in elderly patients on anticoagulation. Neurosurgery.

[23] Kanda Y (2013). Investigation of the freely available easy-to-use software 'EZR' for medical statistics. Bone Marrow Transplant.

[24] Daisuke I, Koji M, Daizoh S (2015). Analysis of pedestrian and cyclist injuries using Japan Trauma Data Bank. Journal of the Japanese Council of Traffic Science.

[25] Akihiro M, Yusuke S, Hiroshi N, Junichi O (2019). Recent trend of severe head injury cases due to road traffic accident: epidemiological features and outcomes in the Japan Neurotrauma Data Bank (Article in Japanese). Neurotraumatology.

[26] Motonobu K, Hiroshi K, Makoto K, Toshiaki H, Takayuki H, Teiji T (2013). Severe head injury and age in Japan Neurotrauma Data Bank: Comparison among Project 1998, 2004 and 2009. Neurotraumatology.

[27] (2025). Cabinet Office: FY2022 Status of Traffic Accidents and Current State of Traffic Safety Measures, FY2023 Plans Regarding the Traffic Safety Measures (White Paper on Traffic Safety in Japan 2023). https://www8.cao.go.jp/koutu/taisaku/r05kou_haku/english/pdf/wp2023.pdf.

[28] Shoji Y, Takashi A, Hidetaka O, Gaku M, Yasuhiro T, Akira F, Hiroyuki Y (2013). Aggressive treatment and patient outcomes for elderly patients with severe head injury: A study of the Head Injury Data Bank (Projects 1998, 2004, 2009) (Article in Japanese). Neurotraumatology.

[29] (2025). Cabinet Office: Annual Report on the Ageing Society (Summary) FY2021. https://www8.cao.go.jp/kourei/english/annualreport/2021/pdf/2021.pdf.

[30] Morganti-Kossmann MC, Satgunaseelan L, Bye N, Kossmann T (2007). Modulation of immune response by head injury. Injury.

[31] Asami I, Kazuo O, Shingo I, Yasuyuki K, Tadahiko S, Hidetada F, Michiaki H (2012). Outcome of patients with multiple trauma associated with head injury -a comparative study with head injury alone. J Jpn Assoc Surg Trauma.

[32] Post A, Hoshizaki TB, Gilchrist MD, Brien S, Cusimano M, Marshall S (2015). Traumatic brain injuries: the influence of the direction of impact. Neurosurgery.

[33] Torimitsu S, Nishida Y, Takano T (2015). Statistical analysis of biomechanical properties of the adult sagittal suture using a bending method in a Japanese forensic sample. Forensic Sci Int.

[34] Toida C, Muguruma T, Gakumazawa M, Shinohara M, Abe T, Takeuchi I, Morimura N (2021). Age- and severity-related in-hospital mortality trends and risks of severe traumatic brain injury in Japan: a nationwide 10-year retrospective study. J Clin Med.

[35] Hosomi S, Sobue T, Kitamura T, Ogura H, Shimazu T (2022). Nationwide improvements in geriatric mortality due to traumatic brain injury in Japan. BMC Emerg Med.

